# The Role of the NF-κB Signaling Pathway in Atherosclerotic Plaque Rupture and Targeted Therapeutic Strategies

**DOI:** 10.3390/biomedicines14010201

**Published:** 2026-01-16

**Authors:** Lihui Yin, Xuehua Wang, Ni Xiong, Jinjie Xiong, Qianyi Liu, Han Li, Yanling Huang, Jiaxi Lv, Yan Wang, Zhaohui Wang

**Affiliations:** 1 Department of Cardiology, Union Hospital, Tongji Medical College, Huazhong University of Science and Technology, Wuhan 430022, China; ylh20022025@163.com (L.Y.); d202382116@hust.edu.cn (X.W.); m202376090@hust.edu.cn (N.X.); xiong_jingjie@163.com (J.X.); qiany_liu@163.com (Q.L.);; 2 Hubei Key Laboratory of Biological Targeted Therapy, Union Hospital, Tongji Medical College, Huazhong University of Science and Technology, Wuhan 430022, China; 3 Hubei Provincial Engineering Research Center of Immunological Diagnosis and Therapy for Cardiovascular Diseases, Union Hospital, Tongji Medical College, Huazhong University of Science and Technology, Wuhan 430022, China

**Keywords:** NF-κB, inflammation, atherosclerosis, pathological angiogenesis, epigenetic regulation, NIK inhibitor

## Abstract

Atherosclerosis (AS) is a disease characterized by chronic vascular wall inflammation and lipid deposition. Although lipid-lowering drugs such as statins have significantly reduced cardiovascular event rates, “residual inflammatory risk” remains a key factor driving disease progression and plaque rupture. As a central regulator of the inflammatory response, the nuclear factor-κappaB (NF-κB) signaling network comprises both canonical pro-inflammatory pathways and functionally more complex non-canonical pathways. Increasing evidence in recent years indicates that abnormal and sustained activation of the non-canonical NF-κB signaling pathway plays a pivotal role in driving plaque rupture. This review first elaborates on the shift in AS strategies from “lipid-lowering” to “anti-inflammatory” approaches, followed by an in-depth analysis of the molecular activation mechanisms of the NF-κB signaling pathway and its distinctiveness in the AS pathological process, along with its epigenetic regulation. It emphasizes how this pathway drives pathological angiogenesis and regulates vascular smooth muscle cell (VSMC) phenotypic switching and macrophage function, thereby forming a vicious cycle that amplifies inflammation and structural damage, ultimately leading to acute cardiovascular events. Finally, we systematically summarize current progress and challenges in drug development targeting the NF-κB pathway (e.g., targeting key kinases like NIK and IKKα), aiming to provide theoretical foundations and future directions for novel therapeutic strategies to stabilize coronary plaques and prevent acute coronary syndromes.

## 1. Introduction

Atherosclerosis (AS), as the primary pathological basis of cardiovascular disease (CVD), remains a major contributor to the persistently high incidence and mortality rates of CVD globally, constituting a significant global disease burden [1]. For a long time, AS has been widely regarded as a disorder of lipid metabolism. Consequently, lipid-lowering therapies, represented by statins, have remained the core strategy and have achieved success in reducing cardiovascular events [2,3]. However, even when lipid levels are well-controlled, patients often exhibit persistently elevated inflammatory markers such as high-sensitivity C-reactive protein (hs-CRP). This persistent inflammatory state is closely associated with atherosclerotic plaque rupture and the occurrence of acute coronary syndromes (ACS). This phenomenon is termed residual inflammatory risk (RIR) [4]. In studies exploring atherosclerosis, the CANTOS trial first compellingly demonstrated that canakinumab, targeting interleukin-1β (IL-1β), significantly reduces cardiovascular event rates, with particularly pronounced effects in patients with reduced hs-CRP levels [5]. In recent years, epidemiological, clinical, and imaging studies have supported the view that inflammation and immune mechanisms are core drivers of AS and its clinical events [6,7]. Current consensus holds that AS is fundamentally a chronic inflammatory disease throughout its entire course [7,8,9], characterized by a cascade of inflammatory responses including inflammatory cells infiltration, cytokine release, and pathological neovascularization within plaques [10]. Inflammatory responses play an indispensable role in the initiation, progression, and eventual rupture of atherosclerotic plaques. In particular, regarding plaque rupture, the persistent presence and abnormal activation of inflammation are key factors leading to increased plaque vulnerability, ultimately resulting in rupture and triggering acute cardiovascular events such as myocardial infarction and stroke [11,12,13,14].

NF-κB signaling pathway is a crucial family of intracellular transcription factors, attracting significant attention due to its central role in regulating diverse physiological and pathological processes including inflammatory reactions, immune responses, cell proliferation, differentiation, and apoptosis [15,16]. The NF-κB signaling pathway is primarily categorized into canonical and non-canonical pathways based on their activation mechanisms and downstream effect [9,17,18,19]. While the canonical NF-κB signaling pathway dominates acute inflammatory responses, recent studies reveal that the non-canonical pathway exerts unique, sustained regulatory functions in chronic inflammatory diseases, particularly in the progression of atherosclerotic plaques [18,19,20,21]. Abnormal activation of the non-canonical NF-κB signaling pathway not only directly promotes inflammatory cytokine expression but may also exacerbate plaque vulnerability through mechanisms such as inducing pathological angiogenesis, ultimately forming a vicious cycle that accelerates plaque rupture [22,23,24,25].

This review aims to summarize the role of the NF-κB signaling pathway in coronary atherosclerotic plaque rupture and explore targeted therapeutic strategies. We will first examine the inflammatory nature of atherosclerosis and its intrinsic link to plaque instability, followed by an in-depth analysis of NF-κB signaling pathways and their epigenetic regulatory mechanisms. Subsequently, this chapter will emphasize the pivotal role of pathological angiogenesis in plaque instability, revealing how the NF-κB pathway drives this process to create a vicious cycle promoting plaque rupture. Finally, we will summarize existing therapeutic strategies targeting the NF-κB pathway to stabilize atherosclerotic plaques. Building upon this foundation, we will identify gaps in current research, providing a theoretical basis for the entry points and innovations of this study.

## 2. Formation of Atherosclerotic Plaques and Inflammatory Mechanisms

### 2.1. Formation of Atherosclerotic Plaques

Atherosclerosis (AS), a leading global cause of cardiovascular disease (CVD) morbidity and mortality, has undergone significant therapeutic evolution over the past few decades. Initial interventions that focused on lipid metabolism have progressively expanded to target inflammatory responses [1,24]. This shift reflects deepening insights into the pathophysiological mechanisms of atherosclerosis. AS is now clearly defined as a chronic inflammatory disease characterized by a cascade of inflammatory events including endothelial dysfunction, inflammatory cell infiltration, cytokine release, and pathological neovascularization within plaques [8,9,10].

The formation of atherosclerotic plaques is a progressive process, often initiated by vascular endothelial injury and dysfunction. Endothelial damage is typically caused by multiple risk factors including hyperlipidemia (particularly ox-LDL), hypertension, hyperglycemia, and smoking [26,27]. Under conditions of endothelial injury and dysfunction, low-density lipoprotein (LDL) enters the arterial wall, leading to lipid deposition within the vessel wall—the initial step in plaque formation [28]. During this process, LDL particles undergo oxidation, forming oxidized low-density lipoprotein (ox-LDL) [29]. As oxidized lipids accumulate, monocytes migrate from the bloodstream to the injured site and differentiate into macrophages. Macrophages transform into foam cells by phagocytosing oxidized lipids, forming the early core of the plaque [30]. Foam cells gradually undergo necrosis, releasing additional lipids that further exacerbate inflammation and plaque instability [31]. Recent studies indicate that inflammation not only causes plaque formation and rupture but also acts as a promoter, potentially accelerating plaque development by altering endothelial cell function. Cytokines (IL-6, TNF-α) play a pivotal role in mediating inflammatory responses and arterial remodeling [32]. Moreover, plaque instability correlates with the structure of its fibrous cap. High-resolution imaging studies have revealed subtle changes in plaque components, indicating that reduced fibrous cap thickness and expansion of the central lipid core are precursors to plaque rupture and acute coronary syndromes [33]. However, despite significant advances in understanding the inflammatory mechanisms of atherosclerosis, our comprehension of atherosclerotic plaque progression remains incomplete. Current research primarily focuses on inflammatory mechanisms and lipid metabolism, while studies on how the plaque microenvironment triggers acute coronary syndromes remain insufficient.

### 2.2. Inflammation-Mediated Atherosclerotic Plaque Rupture

Rupture of vulnerable plaques is the primary cause of acute coronary syndrome (ACS) and ischemic stroke [12,13]. Vulnerable plaques exhibit a series of characteristic pathological features, including a thin, fragile fibrous cap, a large lipid-rich necrotic core, dense inflammatory cell infiltration, and the formation of neovascularization within the plaque [11]. Collectively, these features lead to reduced mechanical strength and increased rupture risk. Among these, the fibrous cap serves as a critical structural barrier separating the plaque core from the bloodstream. Its thickness and integrity are key indicators for assessing plaque stability. When the fibrous cap becomes thin and enriched with inflammatory cells (particularly macrophages), the plaque becomes highly susceptible to rupture [12].

Inflammation directly compromises cap integrity by disrupting the balance between synthesis and degradation of the extracellular matrix (ECM). The primary effector cells—macrophage-activated within plaques by oxidized low-density lipoprotein (ox-LDL)—and cholesterol crystals secrete large amounts of matrix metalloproteinases (MMPs), particularly MMP-1, MMP-8, and MMP-13 (collagenases) as well as MMP-2 and MMP-9 (gelatinases) [34]. These enzymes specifically degrade type I and III collagen in the fibrous cap, leading to its thinning and eventual rupture [35,36]. Concurrently, inflammation severely disrupts the plaque’s repair mechanisms, where vascular smooth muscle cells (VSMCs) serve as the primary cellular source for collagen synthesis and maintenance of fibrous cap strength. Under sustained stimulation by pro-inflammatory cytokines (such as IFN-γ, TNF-α, and IL-1β), VSMCs undergo phenotypic transformation from a contractile to a synthetic or macrophage-like phenotype, resulting in the loss of their collagen-synthesizing capacity [37]. More critically, inflammation induces apoptosis and necrosis in VSMCs, further diminishing matrix production and rendering the plaque “shell” increasingly fragile [38,39].

The expansion of the plaque core is primarily attributed to inflammation-mediated cell death and clearance impairment. In advanced plaques, macrophages undergo apoptosis due to excessive lipid uptake. Under physiological conditions, apoptotic cells are rapidly cleared via efferocytosis. However, in the inflammatory environment of AS, impaired efferocytosis leads to secondary necrosis of apoptotic cells. This releases intracellular toxic substances, enlarging the necrotic core and triggering more intense inflammatory responses [40,41]. Furthermore, neutrophil infiltration and the release of neutrophil extracellular traps (NETs) have been demonstrated to contribute to plaque instability. NETs not only contain cytotoxic histones but also directly activate endothelial cells and promote thrombosis [42,43]. This complex inflammatory cascade is largely regulated by the NF-κB signaling pathway. As a pivotal hub of inflammation, NF-κB induces the transcription of key pro-inflammatory factors including IL-1β, IL-6, and TNF-α, forming a positive feedback loop [44]. Subsequent studies further revealed that NLRP3 inflammasome activation closely coordinates with NF-κB signaling, driving inflammatory progression in plaques by processing mature IL-1β and IL-18 [45,46]. In summary, inflammation comprehensively undermines plaque stability by promoting matrix degradation, inhibiting matrix synthesis, inducing cell necrosis, and impeding inflammatory resolution, constituting the fundamental pathophysiological basis for plaque rupture.

## 3. Role of NF-κB Signaling in the Plaque Microenvironment

NF-κB is a family of transcription factors composed of multiple subunits that play a central regulatory role in various physiological and pathological processes, including immunity, inflammation, cell proliferation, differentiation, and apoptosis [9]. Members of the NF-κB family include RelA (p65), RelB, c-Rel, NF-κB1 (p105/p50), and NF-κB2 (p100/p52), which form homodimers or heterodimers, with the p50/p65 heterodimer being the most prevalent [17]. The activation of NF-κB involves two signaling pathways: the canonical and the non-canonical (alternative) pathway (Table 1) [47].

### 3.1. Rapid Inflammatory Amplification by the Canonical NF-κB Pathway

**Figure 1:**
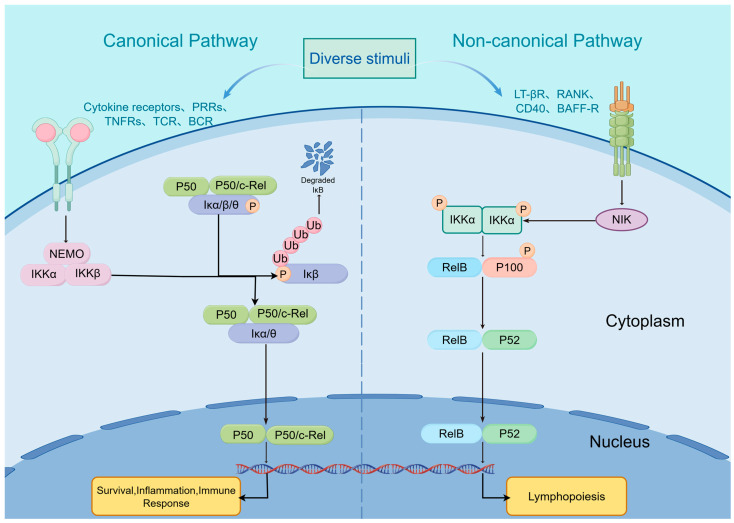
The activation of the canonical and non-canonical NF-κB pathways of stimuli induction toward NF-kB’s nuclear translocation. Created in Figuredraw. Lihui Yin. (2026) https://www.figdraw.com/.

The canonical NF-κB pathway primarily mediates rapid, potent acute inflammatory responses and serves as the initial signal driving the transition from stable to unstable atherosclerotic plaques [9]. Activation of this pathway critically depends on the NEMO (NF-κB Essential Modulator, also known as IKKγ) subunit within the IKK complex (Figure 1). While serving as the first line of defense against pathogen-associated molecular patterns (PAMPs) and damage-associated molecular patterns (DAMPs), this defense mechanism transforms into a core driver destabilizing atherosclerotic plaques within the pathological environment [48].

In the plaque microenvironment, oxidative low-density lipoprotein (ox-LDL), lipopolysaccharide (LPS), and hypoxia activate the classical pathway through Toll-like receptors (TLRs) or cytokine receptors (e.g., IL-1R, TNFR) [49]. This process rapidly induces phosphorylation of the IκB kinase (IKK) complex (comprising IKKα, IKKβ, and NEMO), leading to the ubiquitination and degradation of the inhibitory protein IκBα. Degradation of IκBα releases the bound p65/p50 heterodimer, which rapidly translocates to the nucleus and initiates explosive transcription of pro-inflammatory genes [47]. During the early and progressive stages of atherosclerotic lesions, sustained activation of the canonical NF-κB pathway in endothelial cells directly upregulates the expression of E-selectin, vascular cell adhesion molecule-1 (VCAM-1), and intercellular adhesion molecule-1 (ICAM-1) [50]. These adhesion molecules act as “anchors”, capturing circulating monocytes and T lymphocytes in the bloodstream and promoting their migration into the subendothelium. Furthermore, this pathway induces the release of chemokines such as MCP-1 (CCL2), creating a positive feedback loop that leads to the accumulation of inflammatory cells within the plaque. This represents the cellular basis for plaque enlargement [51,52].

Second, the canonical NF-κB signaling pathway drives M1 macrophage polarization and the production of a “cytokine storm”. Following differentiation into macrophages within the plaque, the canonical NF-κB pathway dominates their polarization toward the pro-inflammatory M1 phenotype [53]. The p65/p50 complex strongly induces transcription of pro-inflammatory cytokines such as TNF-α, IL-1β, IL-6, and IL-12. Notably, TNF-α and IL-1β not only act as effector molecules of inflammation but also re-activate the NF-κB pathway via autocrine or paracrine mechanisms, forming a self-amplifying “inflammatory storm” [54]. This cascade significantly elevates oxidative stress levels within the plaque, leading to endothelial cell apoptosis and compromised barrier function, thereby creating conditions conducive to intraplaque hemorrhage [55]. Ultimately, this triggers transcriptional regulation of matrix metalloproteinases (MMPs) and thinning of the fibrous cap. This represents the most direct mechanism leading to plaque rupture, with the canonical NF-κB pathway serving as a key transcriptional regulator for multiple MMP genes (including MMP-1, MMP-2, MMP-9, and MMP-13) [34].

In the shoulder region of vulnerable plaques, activated macrophages and foam cells massively secrete MMP-9 via the NF-κB pathway. MMP-9 possesses potent gelatinase activity, specifically degrading type IV collagen and elastin in the extracellular matrix, leading to thinning and weakened strength of the protective fibrous cap [36,56]. Concurrently, NF-κB inhibits collagen synthesis in vascular smooth muscle cells (VSMCs) and induces their apoptosis, further impairing the cap’s repair capacity and rendering the plaque highly susceptible to rupture under blood flow shear stress [37]. When the classical NF-κB pathway (p65) is strongly activated, it recruits epigenetic readers such as BRD4 to form massive regulatory hubs on chromatin known as super-enhancers. These super-enhancers synergistically and efficiently drive exponential expression of a comprehensive set of key inflammatory genes (e.g., adhesion molecule VCAM-1, chemokine CCL2), elucidating how NF-κB dynamically remodels super-enhancers to precisely regulate inflammatory responses. This mechanism offers novel therapeutic targets for diseases like atherosclerosis [57].

### 3.2. Sustained Inflammatory Activation by the Non-Canonical NF-κB Pathway

The non-canonical NF-κB pathway, also known as the alternative pathway, exhibits relatively slow and typically sustained activation mechanisms. It primarily participates in lymphoid organ development, immune cell differentiation, maintenance of immune homeostasis, and regulation of specific inflammatory responses [58]. Unlike the canonical pathway, activation of the non-canonical NF-κB pathway does not depend on NEMO (IKKγ) but is strictly controlled by the accumulation of NF-κB-inducing kinase (NIK) at the protein level. Under resting conditions, NIK is ubiquitinated and degraded by the TRAF3-TRAF2-cIAP1/2 complex. Upon pathological stimulation, this complex dissociates, allowing NIK to stabilize and phosphorylate downstream IKKα. This subsequently triggers phosphorylation and ubiquitin-dependent processing of the p100 protein, yielding the mature p52 subunit [59]. p52 forms heterodimers with RelB (p52/RelB) and translocates to the nucleus, initiating transcription of specific target genes [60]. Notably, the non-canonical pathway is not entirely independent from the canonical pathway. The p100 protein itself is a member of the IκB family, capable of sequestering the p65/p50 dimer of the canonical pathway. When activation of the non-canonical pathway leads to the processing of p100 into p52, the sequestered p65 is released, amplifying the inflammatory signal. This “crosstalk” mechanism explains why inflammatory responses often persist in chronic inflammation [61]. TRIM67 negatively regulates TNF-induced NF-κB activation by competitively binding β-TrCP to inhibit IκBα degradation, thereby reducing pro-inflammatory factor production (IL-6, TNF), suggesting its potential role in chronic inflammation [62]. Concurrently, the non-canonical pathway plays crucial roles in regulating B and T cell development, lymph node formation, and maintaining immune tolerance. For example, microRNAs (e.g., miR-223, miR-15a, miR-16) decrease during macrophage differentiation, thereby increasing IKKα expression. This leads to NIK stabilization and p52 accumulation, ultimately suppressing NF-κB-targeted gene expression. This suggests microRNAs may influence specific macrophage responses by regulating the non-canonical NF-κB pathway [63]. Recent studies have developed novel IKKα inhibitors capable of selectively disrupting cellular non-canonical NF-κB signaling, providing pharmacological tools for investigating IKKα and IKKβ functions [64].

### 3.3. Epigenetic Regulation of the NF-κB Signaling Pathway

The activity of the NF-κB signaling pathway is not only finely regulated by post-translational modifications such as protein phosphorylation and ubiquitination [65], but also influenced by complex epigenetic mechanisms, including DNA methylation, histone modifications, and the regulation of non-coding RNAs (ncRNAs) [66]. NF-κB’s transcriptional activity is closely linked to chromatin accessibility. Histone acetylation typically relaxes chromatin structure, facilitating easier binding of transcription factors to DNA and thereby promoting gene expression. NF-κB itself can recruit histone acetyltransferases (HATs) to the promoter regions of its target genes, promoting histone acetylation and subsequently enhancing gene expression. Conversely, histone deacetylases (HDACs) suppress NF-κB’s transcriptional activity [67]. SIRT1, an NAD+-dependent deacetylase, has been found to inhibit NF-κB’s transcriptional activity by deacetylating its subunits (e.g., p65), thereby exerting anti-inflammatory effects [68]. Long non-coding RNAs (lncRNAs), another crucial class of epigenetic regulators, play an increasingly significant role in modulating the NF-κB signaling pathway and are closely associated with atherosclerosis [26]. Defects in the lncRNA MALAT1 in ApoE-/- mice lead to immune dysregulation and atherosclerosis, potentially through enhanced TNF and NOS2 expression in macrophages [27]. The macrophage-enriched lncRNA RAPIA, highly expressed in atherosclerotic lesions and macrophages, inhibits atherosclerotic progression upon suppression, suggesting lncRNA RAPIA may regulate macrophage function by affecting pathways such as NF-κB [69]. Furthermore, Kaszycki and Kim (2025) noted that epigenetic modifications, including DNA methylation, histone modifications, and ncRNAs, significantly influence genes in the NF-κB and NLRP3 inflammasome signaling pathways, offering novel therapeutic strategies for inflammatory diseases [70].

## 4. The Vicious Cycle of NF-κB Signaling-Mediated Inflammation

### 4.1. Vascular Endothelial Injury and Sustained Activation of the NF-κB Signaling Pathway

Vascular endothelial injury triggers recruitment and infiltration of inflammatory cells (e.g., monocytes/macrophages), inducing intense local inflammation. Among these pathways, the NF-κB signaling pathway is significantly activated. As inflammation persists, multiple immune cells (macrophages, lymphocytes) accumulate at the lesion site and release large amounts of cytokines (e.g., IL-1, TNF-α, and IFN-γ) and growth factors [71]. These signals stimulate smooth muscle cells (SMCs) in the vascular wall to migrate from the media to the intima and proliferate in the subintima. SMCs migrating to this region synthesize large amounts of extracellular matrix (primarily collagen), forming a “fibrous cap” overlying the lipid core. The fibrous cap isolates pro-inflammatory and pro-coagulant substances within the plaque, maintaining its stability [72]. In the late stage, foam cells and SMCs within the plaque core undergo apoptosis and necrosis, releasing cellular contents to form an amorphous necrotic core rich in lipids and cellular debris. A plaque characterized by a thin fibrous cap (cap thickness < 65 μm), a large necrotic core (NC area > 30%), and extensive inflammatory cell infiltration is termed a “vulnerable plaque.” Vulnerable plaques are highly susceptible to rupture and subsequent thrombotic events [73]. Within the plaque microenvironment, immunofluorescence confirmed NIK expression in AS plaque microvessels, accompanied by IKKα phosphorylation (Ser176/180) and CXCL12 upregulation. Overexpressed NIK enhances chemokine and cytokine expression (TNF-α, IL-6, IL-12, IL-15, IL-8, MIP-1α/β, CCL3, MCP-1, etc.), thereby initiating immune responses and recruiting immune cells. These immune cells express activators of the non-canonical NF-κB pathway (e.g., LTb, LIGHT, BAFF, CD40L), ultimately forming a persistently activated inflammatory amplification pathway [74].

### 4.2. Intraplaque Hemorrhage Exacerbates NF-κB-Mediated Inflammatory Responses

A close crosstalk mechanism exists between NF-κB and the NLRP3 inflammasome, which is a core component leading to increased vulnerability of plaques. NF-κB first acts as a priming signal, upregulating the expression of NLRP3 protein and pro-IL-1β [45]. Subsequently, upon exposure to “activation signals” such as cholesterol crystals, the NLRP3 inflammasome assembles and activates Caspase-1, cleaving pro-IL-1β into mature IL-1β [46]. Mature IL-1β not only further induces systemic release of IL-6 and CRP but also continuously activates NF-κB through positive feedback loops, forming an “inflammatory storm” [75]. This sustained inflammatory response stimulates macrophages and foam cells to secrete matrix metalloproteinases (MMPs), which degrade collagen and elastin in the fibrous cap of the plaque. This leads to cap thinning, expansion of the necrotic core, and ultimately triggers plaque rupture and thrombosis [36]. Abnormal expression and elevated activity of MMPs are closely associated with atherosclerotic progression and plaque rupture, positioning MMPs as potential biomarkers and therapeutic targets for plaque instability [76]. Specifically, MMP-1, MMP-2, MMP-3, and MMP-9 degrade collagen and elastin within the extracellular matrix (ECM), compromising the structural integrity of the fibrous cap [77,78]. Furthermore, intraplaque hemorrhage is a significant contributor to plaque instability, with ruptured neovasculature being the primary source of such bleeding [14,24].

Vascular endothelial growth factor (VEGF), one of the most prominent known proangiogenic factors, binds to its receptor VEGFR2 to activate downstream signaling pathways, thereby promoting proliferation, migration, and angiogenesis of vascular endothelial cells. Activation of non-NF-κB pathways drives pathological angiogenesis within plaques, forming numerous structurally immature, highly permeable neovessels [74]. Under conditions of high lipid and high shear stress, the VEGF-VEGFR2 signaling pathway regulates angiogenesis, promoting the formation of atherosclerotic vulnerable plaques [79]. In other words, NF-κB drives pathological angiogenesis within atherosclerotic plaques by upregulating the VEGF/VEGFR2 signaling pathway. The formation of new blood vessels, on one hand, exacerbates the infiltration of immune cells and, on the other hand, creates conditions for intraplaque hemorrhage (IPH), ultimately contributing to plaque instability [74]. These neovessels are typically structurally compromised, with thin walls and high permeability, making them highly susceptible to rupture and bleeding. The leaked erythrocytes release heme, which itself is a potent inducer of inflammation and oxidative stress, which can activate pattern recognition receptors like Toll-like receptor 4 (TLR4). This, in turn, strongly activates the canonical NF-κB pathway in macrophages and introduces more inflammatory cells and erythrocyte degradation products, further intensifying the inflammatory response and oxidative stress within the plaque [80]. Concurrently, myeloperoxidase (MPO) release and iron deposition from intraplaque hemorrhage activate the NLRP3 inflammasome to promote IL-1β secretion, further compromising plaque stability and forming a vicious cycle that promotes angiogenesis and plaque instability [81].

### 4.3. Plaque Rupture and Acute Cardiovascular Events

Persistent activation and lipid overload of inflammatory cells (particularly macrophages) within plaques lead to apoptosis and pyroptosis, forming large necrotic cores. The expansion of these necrotic cores further compromises plaque structural integrity, increasing susceptibility to rupture. Simultaneously, intracellular components released by necrotic cells—such as cholesterol crystals and damage-associated molecular patterns (DAMPs)—further activate the NF-κB pathway and NLRP3 inflammasome, intensifying the inflammatory response [82]. Persistent inflammation and activation of the non-canonical NF-κB pathway lead to overexpression and increased activity of matrix metalloproteinases (MMPs). MMPs degrade extracellular matrix components like collagen and elastin within the fibrous cap, causing its thinning and reduced strength [83]. Thinning of the fibrous cap renders it more susceptible to rupture under blood flow shear stress. Once ruptured, exposed plaque contents rapidly activate platelets and the coagulation system, leading to thrombus formation and triggering acute cardiovascular events [73].

The non-canonical NF-κB pathway collectively drives structural instability in atherosclerotic plaques through multiple mechanisms, including inducing chronic, persistent inflammation, promoting fibrin cap degradation, driving pathological angiogenesis, and modulating cell death patterns. Therefore, targeting the non-canonical NF-κB pathway, particularly its upstream kinases (e.g., NIK, IKKα) or downstream effector molecules (e.g., CXCL12), holds promise as an effective therapeutic strategy for stabilizing coronary plaques and preventing acute cardiovascular events.

## 5. Advances on Therapeutic Strategies Targeting the NF-κB

As previously described, the NIK-mediated non-canonical pathway in atherosclerosis has been demonstrated to be closely associated with chronic inflammation in advanced lesions, intraluminal neovascularization, and plaque instability. Studies reveal a significant increase in NIK-expressing endothelial cells within unstable atherosclerotic plaques and coronary lesions associated with myocardial infarction [74]. This indicates that NIK activation correlates with plaque risk phenotypes (e.g., high lipid content and inflammatory cell infiltration) and the occurrence of clinical events. Given the central role of the NF-κB pathway, particularly NIK, in atherosclerotic inflammation, targeting this pathway is considered a highly promising therapeutic strategy (Table 2) [84].

### 5.1. Inhibition of the Overall NF-κB Signaling Pathway

The NF-κB signaling pathway, as one of the core regulatory hubs of inflammatory responses, holds promise as a key therapeutic target for various inflammation-related diseases [85]. Drug development targeting the NF-κB pathway primarily focuses on inhibiting IKK kinase activity, blocking NF-κB nuclear translocation, and modulating its upstream activation signals. The IKK kinase complex represents a critical node in NF-κB pathway activation, with IKKβ playing a dominant role in the canonical pathway [18,86]. Consequently, developing IKK inhibitors constitutes a key strategy for targeting the NF-κB pathway. For instance, inhibiting the upstream IKK complex (particularly IKKβ) effectively blocks classic NF-κB pathway activation, thereby reducing inflammatory cytokine production and diminishing atherosclerotic plaque size in animal models [87]. Small-molecule inhibitors like BAY11-7082 block IKKβ, suppressing NF-κB phosphorylation (50% reduction in p65) and STAT3 activation (60% decrease in pSTAT3), downregulating NLRP3 inflammasome activity and reducing IL-1β secretion to lower inflammatory risk. However, systemic immunosuppression remains a potential concern [85,88].

Natural products and their derivatives, characterized by multi-targeted effects and low toxicity, have garnered significant attention in NF-κB pathway inhibitor development. For instance, resveratrol alleviates cardiovascular inflammation by inhibiting NF-κB, but its low bioavailability necessitates the development of nanoformulations or polymer derivatives (e.g., Gnetin-C dimer) to enhance bioavailability [89]. Corydaline improves vitamin D3/high-cholesterol diet-induced atherosclerosis in rats by regulating miR-34a and the Wnt5a/Ror2/ABCA1/NF-κB pathway [90]. 4-Methoxycinnamic acid hydroxycoumarin exerts anti-atherosclerotic effects by inhibiting NF-κB signaling and foam cell formation [91]. Synephrine regulates macrophage foam cell formation and atherosclerosis by inhibiting Piezo1-mediated MAPK/NF-κB pathway [87]. Sodium danesin suppresses macrophage inflammation and alleviates atherosclerosis via miR-200a-3p/MEKK3/NF-κB pathway [92]. Furthermore, papain exerts anti-atherosclerotic effects by inhibiting MPA-induced foam cell formation through regulating MAPK and PI3K/Akt-NF-κB pathways [93]. These studies provide abundant resources for developing natural product-based NF-κB inhibitors.

### 5.2. Specific Targeting of NIK and Non-Canonical Pathways

Given that the canonical NF-κB pathway also plays a crucial role in maintaining normal immune function, broad inhibition of this pathway may lead to side effects such as immunosuppression. Therefore, specifically targeting the regulation of the non-canonical pathway involved in chronic inflammation, particularly its key kinase NIK, may represent a more precise and safer strategy. Research indicates that targeting the non-canonical NF-κB signaling pathway may hold therapeutic potential for patients with atherosclerosis and cardiovascular disease. By inhibiting NIK, signals leading to chronic vascular inflammation and plaque instability can be precisely blocked, potentially establishing a novel therapy for plaque stabilization and cardiovascular event prevention. Tang (2023) conducted computational simulations to analyze the binding mode of NIK inhibitors, providing theoretical guidance for developing more efficient non-canonical NF-κB pathway inhibitors [94]. The small-molecule inhibitor B022 (Ki = 4.2nM) specifically suppresses NIK kinase activity, inhibiting the NIK/p52 pathway while downregulating pro-inflammatory factors such as TNF-α, IL-6, and iNOS expression, thereby alleviating oxidative stress [95]. Small-molecule inhibitors targeting NIK demonstrate significant therapeutic potential in inflammation, tumors, and metabolic diseases by regulating the non-canonical NF-κB pathway [96]. Future efforts should focus on preclinical safety evaluation, exploration of novel drug scaffolds, and precise indication selection to advance their clinical application.

### 5.3. Emerging Therapies

Novel drug delivery systems, such as nanoparticles, are also employed to enhance the targeting and bioavailability of NF-κB inhibitors at plaque sites. Utilizing VHPK-PLGA@COL nanoparticles to deliver colchicine achieved targeted delivery to inflamed endothelial cells, significantly enhancing anti-atherosclerotic effects [97]. Statin-loaded recombinant high-density lipoprotein (rHDL) nanoparticles can deliver statins directly to plaques, effectively suppressing plaque inflammation. These advanced delivery technologies hold promise for overcoming the systemic side effects of traditional drugs and enabling more precise targeted therapy [2]. Sigala et al. identified a critical link between basic fibroblast growth factor (bFGF) and atherosclerotic plaque instability mediated by the NF-κB pathway. Their study demonstrated that bFGF administration enhances NF-κB transcriptional activity through the specific phosphorylation of the RelA/p65 subunit at Ser536. This activation subsequently upregulates iNOS and MMP-9, both of which are key drivers of plaque destabilization. Crucially, in human carotid plaque specimens, elevated bFGF expression was significantly associated with unstable plaques and symptomatic patients, correlating strongly with increased nuclear localization of activated NF-κB [98].

Long non-coding RNAs (lncRNAs), acting as epigenetic regulators of the NF-κB pathway, are emerging as novel therapeutic targets. Studies reveal that NEAT1 promotes foam cell formation and inflammatory responses via the miR-342-3p/CD36 axis, while MALAT1 promotes plaque instability via the miR-330-5p/NF-κB axis, and siRNA knockdown of MALAT1 alleviates inflammatory responses [66]. Therefore, deciphering the specific roles of lncRNAs in macrophage subpopulations to develop precise methods for regulating the NF-κB pathway holds significant value. Furthermore, in immune checkpoint inhibitor-associated myocarditis, targeting α-myosin-specific T cells alleviates NF-κB-driven inflammation [85].

Collectively, these studies reveal the broad prospects of targeting the NF-κB pathway in atherosclerosis therapy. From kinase inhibitors to natural products, novel delivery systems, and epigenetic regulation, they provide multidimensional options for exploring new strategies to stabilize plaques.

**Table 2:** Current therapies targeting the NF-κB signaling pathway.

Category	Modulator	Disease	Side Effect
NF-κB Inhibitors	BAY 11-7082	Cancer; inflammatory diseases;	Inhibition on the translocation of p65, AP-1, IRF3, and STAT-1 [88]
	Pyrrolidine dithiocarbamate (PDTC)	Inflammatory disease especially AS	Activation of p38 MAPK and JNK; VSMC growth inhibition [99]
	B022	Liver injury and chronic inflammation	Inhibition on NIK induced p100-to-p52 processing, and the expression of inflammatory cytokines and iNOS genes [95]
Natural compounds	Synephrine	AS	inhibiting Piezo1-mediated MAPK/NF-κB pathway [87]
	Sodium danesin	Inflammatory disease; AS	miR-200a-3p/MEKK3/NF-κB pathway [92]
	papain	AS	inactivating the MAPK and PI3K/Akt-NF-κB pathways [93]
	Resveratrol	Cancer; inflammatory diseases;	Blocks IKK activity and suppresses TNF-α-induced NF-κB activation [100]
Nc RNAs	Mir-181a-5p/3p	Vascular inflammation and AS	Alleviation of atherosclerotic plaque formation; decrease in pro-inflammatory gene expression; decrease in infiltration of macrophage, leukocyte and T cell into the lesions; targeting TAB2 and NEMO [101]
	Circ-Sirt1	Cardiovascular diseases	Inhibition on inflammatory phenotypic switching of VSMC and neointimal hyperplasia; impeding NF-κB translocation and its binding to DNA [102]

## 6. Discussion

Although existing research has profoundly elucidated the critical role of NF-κB signaling in atherosclerotic inflammation and plaque instability, and explored multiple therapeutic strategies targeting NF-κB, significant research gaps and challenges remain.

First, the specific mechanisms of non-canonical NF-κB signaling in atherosclerosis remain incompletely elucidated. While the non-canonical pathway is known to function in chronic inflammation and immune homeostasis, and studies have begun to examine its involvement in atherosclerosis [64], deeper mechanistic research is needed to understand how it precisely regulates inflammatory cell function, extracellular matrix remodeling, and pathological angiogenesis within the plaque microenvironment, thereby influencing plaque stability. Specifically, the synergistic or antagonistic interactions between the non-canonical pathway and the canonical pathway in the atherosclerotic pathological process, as well as their differentiated functions in distinct cell types (e.g., macrophages, endothelial cells, smooth muscle cells), require systematic elucidation.

Second, the molecular mechanisms underlying the vicious cycle between the non-canonical NF-κB pathway and pathological angiogenesis require further exploration. While it has been demonstrated that non-canonical NF-κB promotes angiogenesis and that pathological angiogenesis in turn exacerbates plaque inflammation and instability, a comprehensive understanding of how the non-canonical NF-κB pathway specifically drives pathological angiogenesis within plaques, along with the precise molecular nodes and regulatory networks of this “vicious cycle,” remains elusive. For instance, whether the non-canonical NF-κB pathway promotes angiogenesis by regulating specific proangiogenic factors (such as members of the VEGF family or their receptor subtypes) or through phenotypic transformation of vascular endothelial cells, and its role in the structural abnormalities and functional impairments of neovasculature, remain unresolved questions.

Furthermore, the role of super-enhancers in regulating pathological angiogenesis through non-canonical NF-κB pathways remains understudied. Epigenetic regulation, particularly gene expression modulation mediated by super-enhancers, has been demonstrated to exert potent effects on the transcription of NF-κB target genes [103]. However, few studies have focused on how the non-canonical NF-κB pathway reshapes the super-enhancer landscape to regulate gene expression associated with pathological angiogenesis, ultimately affecting atherosclerotic plaque stability. Deepening our understanding of this epigenetic regulatory mechanism may reveal novel roles for the non-canonical NF-κB pathway in atherosclerosis and provide a theoretical basis for developing epigenetically targeted therapeutic strategies.

## Figures and Tables

**Table 1:** The distinct characteristics of the two primary NF-κB signaling cascades.

Feature	Canonical Pathway	Non-Canonical Pathway
Active Dimer	p50/p65 (RelA)	p52/RelB
Key Kinase	IKKβ (requires NEMO)	NIK and IKKα (NEMO independent)
Inhibitor Degradation	IκBα	p100 to p52
Triggers	TNF-α, IL-1β, LPS	CD40L, BAFF, LTβ
Physiological Role	Innate immunity, acute inflammation, cell survival	Lymphoid organ development, adaptive immunity

## Data Availability

No new data were created or analyzed in this study.

## Abbreviations

The following abbreviations are used in this manuscript:

AS	Atherosclerosis
NF-κB	Nuclear Factor-kappa B
CVD	Cardiovascular disease
VSMC	Vascular smooth muscle cell
RIR	Residual inflammatory risk
ACS	Acute coronary syndromes
hs-CRP	High-sensitivity C-reactive protein
IPH	Intraplaque hemorrhage
ECM	Extracellular matrix
ox-LDL	Oxidized low-density lipoprotein
LDL	Low-density lipoprotein
MMPs	Matrix metalloproteinases
NETs	Neutrophil extracellulartraps
NEMO	NF-Κb Essential Modulator
PAMPs	Pathogen-associated molecular patterns
DAMPs	Damage-associated molecular patterns
TLRs	Toll-like receptors
LPS	Lipo-polisaccharide
VCAM-1	Vascular cell adhesion molecule-1
ICAM-1	Intercellular cell adhesion molecule-1
NIK	NF-Κb-inducing kinase
IKK	IκB kinase
VEGF	Vascular endothelial growth factor
